# Childhood adversity is associated with hospitalisations and survival following external causes and non-communicable diseases: a 46-year follow-up of a Stockholm birth cohort

**DOI:** 10.1136/jech-2022-219851

**Published:** 2023-02-03

**Authors:** Josephine Jackisch, Ylva B Almquist

**Affiliations:** 1 Department of Public Health Sciences, Stockholm University, Stockholm, Sweden; 2 Max Planck Institute for Demographic Research, International Max Planck Research School for Population, Health and Data Science, Rostock, Germany

**Keywords:** COHORT STUDIES, MORTALITY, Life course epidemiology, MORBIDITY

## Abstract

**Background:**

Childhood adversity indicated by involvement with child welfare services (ICWS) is associated with increased risks of disease and injuries in young adulthood. It is yet unknown whether such risks are limited to external causes and mental and behavioural disorders or whether they extend beyond early adulthood and to non-communicable diseases (NCDs) with later onset. Moreover, it has not been explored whether ICWS associates with decreased survival prospects following hospitalisation.

**Methods:**

Based on prospective data for a 1953 Stockholm birth cohort (n=14 134), ICWS was operationalised distinguishing two levels in administrative child welfare records (ages 0–19; ‘investigated’ and ‘placed’ in out-of-home care (OHC)). Hospitalisations and all-cause mortality (ages 20–66) were derived from national registers. Hospitalisation records were categorised into external causes and NCDs, and nine subcategories. Negative binomial regression models were used to estimate differences in hospitalisation risks between those with and without experiences of ICWS and Cox survival models to estimate mortality after hospitalisation.

**Results:**

Placement in OHC was associated with higher risks of hospitalisation due to external causes and NCDs and all investigated subcategories except cancers. Risks were generally also elevated among those investigated but not placed. ICWS was further linked to higher mortality risks following hospitalisation.

**Conclusion:**

Differential risk of morbidity and differential survival may explain inequalities in mortality following childhood adversity. We conclude that the healthcare sector might play an important role in preventing and mitigating the elevated risks of externally caused morbidity, disease and premature mortality observed among those with a history of ICWS.

**What is already known**

People with experiences of involvement with child welfare services (ICWS) have often high levels of adverse childhood experiences and experience higher risks of hospitalisation in adulthood due to external causes as well as mental and behavioural disorders. As most previous prospective studies have ended their follow-ups by early adulthood, it remains unclear whether there are also increased risks for other non-communicable diseases with a later onset and whether childhood adversity is linked to differences in survival after onset of disease.

**What this study adds**

This study demonstrates that adults with experiences of ICWS generally have higher risks of being hospitalised due to external causes and non-communicable diseases. Following an initial hospitalisation, they have lower chances of survival.

**How this study might affect research, practice or policy**

The findings highlight several possible entry points for policy and intervention, which include preventing experiences of childhood adversity, mitigating health-related consequences of such experiences and improving healthcare outcomes in this vulnerable group of individuals.

## Introduction

Experiences of childhood adversity are highly prevalent and result in high costs not only to the individual but also to all of society.^1 2^ Therefore, childhood adversity constitutes an important public health concern.^3^ Individuals who experience involvement with child welfare services (ICWS) carry an exceptionally high burden of adverse childhood experiences.^4 5^ Indeed, it has been consistently demonstrated that ICWS is associated with an increased risk of premature mortality.^6–8^ However, the pathways underlying these differences in mortality are not well known. The current study explores two potential explanations: differential risk of hospitalisation due to external causes and non-communicable diseases (NCDs) and differential survival prospects following such hospitalisations. Such an inquiry may lead us to better understand the healthcare sector’s role in mitigating the risk of mortality among individuals with experiences of childhood adversity. Individuals at risk can be identified early through ICWS, which might guide the development of appropriate interventions.

Theoretically, experiences of childhood adversity—and, particularly, those so severe that they lead to ICWS—can be assumed to ‘get under the skin’, changing the epigenome and other biological and psychological response systems.^9 10^ This inscription of social experiences within the body, from in-utero to death, is sometimes referred to as ‘embodiment’.^11^ Through the process of embodiment, those exposed to childhood adversity might be more susceptible to disease development or more likely to experience more severe consequences following disease (including death).

Thus far, morbidity among individuals with experiences of ICWS has primarily been examined in terms of external causes or mental and behavioural disorders.^12–14^ Previous prospective longitudinal studies have, for example, noted increased risks for developing anxiety, depression, substance use disorders and self-harm^13 15–19^ up until early midlife among those placed in out-of-home care (OHC; foster family or institutional care).

Evidence is scarcer concerning risks of diseases with a later age at onset, including cardiovascular diseases (CVDs), cancers, respiratory diseases and diabetes. Alongside mental and behavioural disorders, they represent the main types of NCDs with an extensive individual and societal burden. These five subcategories—most of which are considered preventable—are together estimated to account for 90% of deaths in the European region.^20^

Looking into the broader literature on childhood adversity, retrospective studies have indicated increased risks of developing NCDs among those with adverse childhood experiences.^2 21–27^ There is, however, a paucity of prospective studies. Those that do exist tend to show inconsistent results, for example, regarding the risks for cancer and diabetes, which might be due to relatively limited follow-ups.^21 28–30^

### Study aims

With the aim to explore the role of childhood adversity in relation to hospitalisation risks and survival prospects following hospitalisation, the present study draws on longitudinal prospective data from a Swedish cohort born in 1953 (n=14 134). Childhood adversity is approximated by experiences of ICWS (ages 0–19). Two groups of ICWS are differentiated, indicating the severity of ICWS experiences: individuals who were investigated but not placed, and individuals for whom the involvement resulted in placement in OHC. Following an outcome-wide approach to epidemiology,^31^ we include hospitalisations due to external causes and NCDs as well as all-cause mortality (ages 20–66). More specifically, this study investigates the following hypotheses:

1. Individuals with experiences of ICWS are at higher risk of being hospitalised due to external causes (including self-harm) and NCDs (including mental and behavioural disorders, CVDs, cancers, respiratory diseases and diabetes) when compared with individuals without experiences of ICWS.
2. Individuals with experiences of ICWS have a shorter time to death after initial hospitalisation due to external causes and NCDs compared with their majority population peers.
3. The relationships in hypotheses 1 and 2 follow a gradient that reflects the severity of ICWS experienced.

## Methods

### Study population

The data material is the Stockholm Birth Cohort Multigenerational study (SBC Multigen). The cohort is defined as all children born in 1953 and living in the greater Stockholm metropolitan area in 1963, who could be probability matched to follow-up data from nationwide registers (n= 14,608).^32^ In this study, we restricted the analysis to those who did not die or emigrate without returning before age 20 (n=14,509). The analytical sample includes everyone without missing values on the covariates (n=14,134). Missing observations in family education at age 10 were retained as a separate category.

### Measurements

#### Involvement with child welfare services

In this cohort, ICWS has been shown to be a viable proxy for childhood adversity because of its predictive power for long-term health outcomes such as premature mortality.^7^ Other prospectively measured indicators of childhood adversity (e.g., accumulative experiences of household dysfunction) do not independently of ICWS predict mortality or educational attainment.^33^

The SBC Multigen contains child welfare records from municipal social registers covering the period between 1953 and 1972 (ages 0–19). Besides placement in OHC, these data include referrals to child welfare services that did not result in the child being taken out of the family but rather cases that were dismissed or resulted in warnings or in-home services. This offers an interesting alternative comparison group, encompassing individuals who have displayed signs of adversity severe enough to come to the attention of the child welfare services but not severe enough to justify a placement in OHC. Based on this, we distinguished three mutually exclusive ICWS groups following the severity of ICWS: (1) those without any record in the child welfare register (‘without ICWS’; who are used as reference group in the main analysis), (2) child welfare investigations not resulting in OHC placement (‘investigated’), (3) placement in OHC, including foster and institutional care (‘placed’). The highest order took precedence in coding these groups.

#### Hospitalisation

From the Swedish National Patient Register, we derived data on in-patient care between 1973 and 2019 (ages 20–66), that is, cases of disease that required hospitalisation (at least one overnight stay at the hospital). A total of 11 cause groups were selected based on both primary and secondary diagnoses and classified according to the 10th revision of the International Classification of Diseases and the corresponding codes in the eighth and ninth revisions (see online supplemental table 1). We identified two major cause groups: external causes and NCDs. External causes include hospitalisations due to causes outside the body, for example, injuries, poisoning, accidents, violent assaults. We separated out the subcategory of (intentional and unintentional) self-harm. Within NCDs, we distinguished five subcategories: CVDs, cancers, respiratory diseases, diabetes and mental and behavioural disorders. Under mental and behavioural disorders, we further separate three subcategories: anxiety, depression and substance use disorders. Besides those subcategories, NCDs as an overall category also include in situ and benign neoplasms; diseases of the blood; other endocrine and metabolic disorders; musculoskeletal disorders; diseases of the nervous, digestive or genitourinary system and diseases of the eye, ear and skin.

10.1136/jech-2022-219851.supp1 Supplementary data

#### Death

Mortality data were drawn from the National Cause of Death Register as death by any cause between 1 January 1973 and 31 December 2019 (ages 20–66). Since chronic diseases tend to co-occur, cause-specific mortality might include uncertainty in the registered causes of death. For this reason and to ensure sufficient statistical power, we preferred using all-cause mortality.

#### Covariates

ICWS is associated with the socioeconomic conditions of the family, and such factors have also been shown to influence adulthood morbidity and mortality. Therefore, we included the following covariates: born out of wedlock; mother’s age at birth; occupational status of family at birth (usually assessed through father’s occupation but, if unavailable, based on the mother’s occupation) and family education at age 10.

#### Sex

All analyses are reported separately for men and women, based on the biological sex assigned at birth.

#### Statistical analysis

Follow-up started on 1 January 1973 and ended at death, emigration or end-of follow-up on 31 December 2019. Within this observation window, 519 people got censored at the date of emigration, and 1635 people died.

#### Risk of hospitalisation

We analysed hospitalisation between ages 20 and 66 for each major cause group and subcategory, stratified by sex and ICWS group. First, we calculated hospitalisation prevalence (expressed as %), deriving 95% CIs from predicted margins based on logistic regression analysis. Next, we calculated incidence rates of hospitalisation per 1000 person-years at risk. Incidence measures the occurrence of new cases during a certain period and, thus, signifies a person’s probability of being hospitalised with a disease.

To further examine the first hypothesis, we conducted multivariate modelling to predict frequencies of hospitalisation within each cause group (thereby taking repeated hospitalisations into account). Model fit statistics for the count variable were derived for the covariate-adjusted Poisson, zero-inflated Poisson and negative binomial models (online supplemental table 2). The negative binomial models fit the data best for all outcomes and were, thus, used to examine the differences in the risk of hospitalisation by ICWS groups. We report adjusted incidence rate ratios (IRRs) with 95% CIs.

#### Time to death

The second hypothesis concerns differential survival after hospitalisation. To investigate this, we used Cox survival models estimating the chances of survival on a calendar time scale from the date of the first cause-specific hospitalisation to death from all causes. The few tied failure times were dealt with by the Efron method. We adjusted these models for covariates and for age at first cause-group-specific hospitalisation, reporting HRs over the follow-up period with 95% CIs.

In order to counteract the likelihood of false-positive chance findings due to multiple outcomes, we used Bonferroni correction to correct α (the significance level considered) to <0.004. However, in the tables, we reported CIs at a 95% level to not confuse readers, whereas p<0.004 is marked with a bold face. Data management was conducted in R (V.4.1.0), and descriptives and analyses were made in Stata (V.17.0) using commands ‘nbreg’ and ‘stcox’. Plots were produced in R.

## Results

Characteristics of the study population are shown in table 1. ICWS was relatively prevalent in the cohort: 26.8% of men and 14.5% of women had such experiences in ages 0–19. The prevalence of OHC was 9.2% among men and 8.4% among women. Those with experiences of ICWS were more likely to have been born out of wedlock, have teenage mothers, have fathers with a manual occupational background and were less likely to have parents with secondary or postsecondary educational degrees.

**Table 1:** Sample characteristics, stratified by sex and ICWS group

	Men				Women			
	Without ICWS	Investigated	Placed	Total	Without ICWS	Investigated	Placed	Total
	%	%	%	%	%	%	%	%
Born out of wedlock (yes)	4.32	7.91	20.88	6.55	4.37	12.80	23.02	6.45
Mother’s age at birth								
Teenager	3.66	4.89	10.23	4.51	3.54	5.92	9.45	4.18
20–35 years of age	80.43	80.03	75.57	79.89	80.66	78.20	75.77	80.10
>35 years of age	15.91	15.08	14.20	15.60	15.79	15.88	14.78	15.71
Occupational status of father at birth								
Non-manual worker	57.25	39.61	34.52	52.03	55.01	38.63	33.68	52.22
Manual worker	42.75	60.39	65.48	47.97	44.99	61.37	66.32	47.78
Family education at age 10								
Primary	64.23	82.48	87.36	69.60	66.27	81.75	87.46	68.99
Secondary	19.14	9.78	7.10	16.37	18.17	9.00	7.73	16.74
Tertiary	10.92	3.99	2.41	8.91	10.06	3.32	1.20	8.90
Missing	5.71	3.75	3.13	5.12	5.50	5.92	3.61	5.37
Total, row per cent	73.2	17	9.2	100	85.5	6.1	8.4	100
Total, n	5273	1227	704	7204	5926	422	582	6930

ICWS, involvement with child welfare services.

Table 2 describes the absolute prevalence and incidence rates of hospitalisation. For all subcategories, men and women placed in OHC have higher prevalence and incidence rates than those without experiences of ICWS. The investigated children’s estimates fall between those two groups with few exceptions. Moreover, we found a generally higher median number of hospitalisations (frequency) and a lower median age at first hospitalisation (onset) for both ICWS groups (‘investigated’ and ‘placed’), except for cancers and respiratory diseases (online supplemental table 3).

**Table 2:** Hospitalisations in ages 20–66: prevalence (%) and incidence rates (hospitalisations per 1000 person-years), stratified by sex and ICWS group

	Hospitalisations among men ages 20–66 (n=7204)						Hospitalisations among women ages 20–66 (n=6930)					
	Without ICWS (n=5273)		Investigated (n=1227)		Placed (n=704)		Without ICWS (n=5926)		Investigated (n=422)		Placed (n=582)	
	Estimate	(95% CI)	Estimate	(95% CI)	Estimate	(95% CI)	Estimate	(95% CI)	Estimate	(95% CI)	Estimate	(95% CI)
External causes (n cases=4448)												
Prevalence	30.5	(29.3 to 31.7)	44.0	(41.2 to 46.8)	46.0	(42.3 to 49.7)	27.3	(26.1 to 28.4)	34.6	(30.1 to 39.1)	40.0	(35.5 to 43.5)
Incidence rate	7.7	(7.4 to 8.1)	12.6	(11.6 to 13.7)	14.5	(13.0 to 16.1)	6.6	(6.3 to 7.0)	9.0	(7.6 to 10.5)	10.5	(9.3 to 12.0)
Self-harm (n cases=504)												
Prevalence	2.0	(1.7 to 2.4)	6.4	(5.1 to 7.8)	8.9	(6.8 to 11.1)	2.8	(2.4 to 3.2)	7.3	(4.9 to 9.8)	10.3	(7.8 to 12.8)
Incidence rate	0.5	(0.4 to 0.6)	1.5	(1.2 to 1.8)	2.3	(1.8 to 2.9)	0.6	(0.5 to 0.7)	1.7	(1.2 to 2.4)	2.4	(1.9 to 3.1)
NCDs (n cases=10097)												
Prevalence	67.4	(66.1 to 68.7)	76.0	(73.7 to 78.4)	78.8	(75.8 to 81.9)	71.7	(70.7 to 72.8)	78.0	(74.0 to 81.9)	84.3	(81.4 to 87.3)
Incidence rate	22.2	(21.5 to 22.9)	27.8	(26.1 to 29.6)	35.0	(32.3 to 38.0)	26.6	(25.8 to 27.4)	35.8	(32.2 to 39.9)	42.1	(38.6 to 46.0)
Mental and behavioural disorders (n cases=2013)												
Prevalence	11.3	(10.3 to 12.1)	23.6	(21.3 to 26.0)	37.1	(33.5 to 40.6)	10.2	(9.4 to 10.9)	21.8	(17.9 to 25.7)	29.9	(26.2 to 33.6)
Incidence rate	2.6	(2.4 to 2.8)	5.8	(5.2 to 6.5)	10.8	(9.6 to 12.2)	2.3	(2.2 to 2.5)	5.5	(4.5 to 6.8)	8.0	(6.9 to 9.2)
Anxiety (n cases=509)												
Prevalence	2.6	(2.2 to 3.0)	4.4	(3.4 to 5.5)	8.5	(6.4 to 10.6)	3.2	(2.7 to 3.6)	5.0	(2.9 to 7.1)	8.4	(6.2 to 10.7)
Incidence rate	0.6	(0.5 to 0.7)	1.0	(0.8 to 1.3)	2.0	(1.6 to 2.6)	0.7	(0.6 to 0.8)	1.1	(0.8 to 1.7)	2.0	(1.5 to 2.6)
Depression (n cases =596)												
Prevalence	3.1	(2.6 to 3.6)	4.6	(3.4 to 5.7)	8.9	(6.8 to 11.1)	3.8	(3.3 to 4.2)	6.2	(3.9 to 8.5)	11.0	(8.5 to 13.5)
Incidence rate	0.7	(0.6 to 0.8)	1.1	(0.8 to 1.4)	2.3	(1.8 to 2.9)	0.8	(0.7 to 1.0)	1.5	(1.0 to 2.1)	2.5	(2.0 to 3.2)
Substance use disorders (n cases=1176)												
Prevalence	5.8	(5.2 to 6.5)	20.4	(18.1 to 22.6)	28.4	(25.1 to 31.7)	4.0	(3.5 to 4.5)	15.4	(12.0 to 18.8)	20.3	(17.0 to 23.5)
Incidence rate	1.3	(1.2 to 1.5)	4.9	(4.4 to 5.6)	7.9	(6.9 to 9.0)	0.9	(0.8 to 1.0)	3.6	(2.9 to 4.6)	5.0	(4.2 to 6.0)
CVDs (n cases=3725)												
Prevalence	29.0	(27.8 to 30.2)	35.9	(33.3 to 38.6)	33.8	(30.3 to 37.3)	21.1	(20.1 to 22.2)	27.3	(23.0 to 31.5)	28.4	(24.7 to 32.0)
Incidence rate	6.8	(6.5 to 7.1)	8.7	(8.0 to 9.5)	8.6	(7.6 to 9.7)	4.8	(4.6 to 5.1)	6.3	(5.3 to 7.6)	6.7	(5.8 to 7.8)
Cancers (n cases=1942)												
Prevalence	11.5	(10.6 to 12.3)	12.2	(10.4 to 14.1)	13.5	(11.0 to 16.0)	15.7	(14.7 to 16.6)	16.8	(13.3 to 20.4)	16.7	(13.6 to 19.7)
Incidence rate	2.5	(2.3 to 2.7)	2.7	(2.3 to 3.2)	3.2	(2.6 to 3.9)	3.5	(3.3 to 3.7)	4.0	(3.2 to 5.0)	3.9	(3.2 to 4.7)
Respiratory diseases (n cases=1630)												
Prevalence	10.1	(9.3 to 10.9)	14.3	(12.4 to 16.3)	15.9	(13.2 to 18.6)	10.8	(10.0 to 11.6)	13.0	(9.8 to 16.2)	21.1	(17.8 to 24.5)
Incidence rate	2.3	(2.1 to 2.5)	3.4	(3.0 to 4.0)	4.0	(3.3 to 4.8)	2.4	(2.3 to 2.6)	3.2	(2.5 to 4.1)	5.2	(4.4 to 6.2)
Diabetes (n cases=624)												
Prevalence	4.7	(4.2 to 5.3)	6.8	(5.4 to 8.3)	6.7	(4.8 to 8.5)	3.2	(2.7 to 3.6)	4.5	(2.5 to 6.5)	6.0	(4.1 to 7.9)
Incidence rate	1.1	(1.0 to 1.2)	1.5	(1.2 to 1.9)	1.6	(1.2 to 2.1)	0.7	(0.6 to 0.8)	1.0	(0.7 to 1.6)	1.3	(0.9 to 1.8)

CVDs, cardiovascular diseases; ICWS, involvement with child welfare services; NCDs, non-communicable diseases.

**Figure 1:**
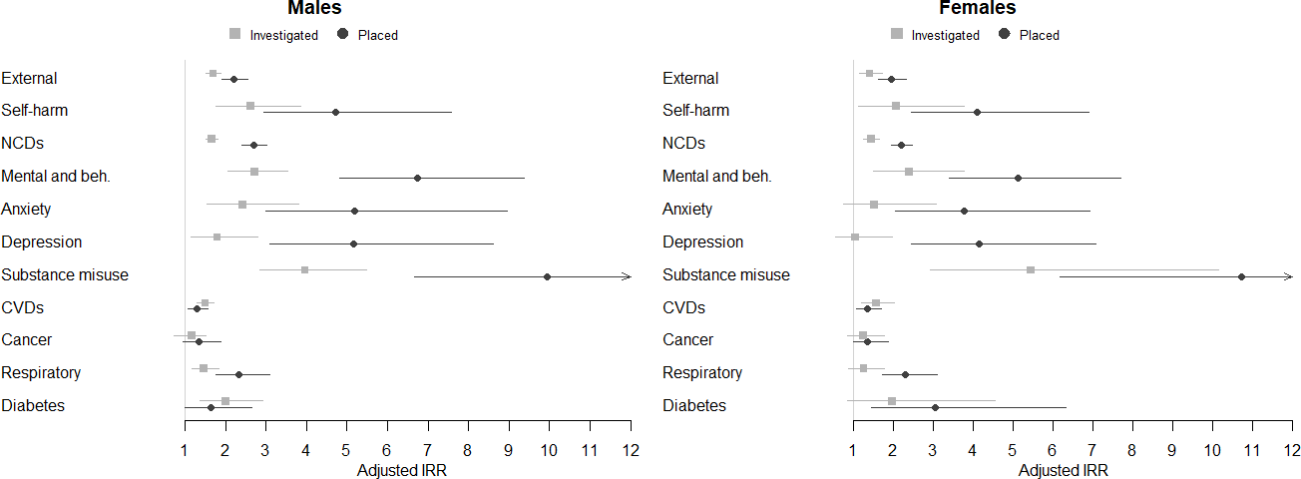
The association between ICWS and hospitalisation, stratified by sex and cause group. Results from the adjusted negative binomial regression models. ‘Without ICWS’ is the reference group (IRR=1.00). All models are adjusted for being born out of wedlock, mother’s age at birth, occupational status at birth, and family education at age 10. CVDs, cardiovascular diseases; IRR, incidence rate ratio; NCDs, non-communicable diseases.

The results from the adjusted negative binomial regression of experiences of ICWS on cause-group-specific hospitalisation are plotted in figure 1. Individuals with ICWS were more likely to be hospitalised due to external causes (IRRs ranging from 1.7 to 2.2) and NCDs (IRRs: 1.7–2.7). Hospitalisation risks were particularly pronounced in the subcategory of mental and behavioural disorders (IRRs: 2.7–6.7), with those who experienced ICWS being more likely to be hospitalised due to substance use disorders (IRRs: 4.0–10.7) and anxiety (IRRs: 2.4–5.2). They also have some increased risks of hospitalisation due to self-harm (IRRs: 2.6–4.7), CVDs (IRRs: 1.3–1.6) and respiratory diseases (IRRs: 1.3–2.3). Estimates were less consistent for diabetes (IRRs: 1.6–3.0) and statistically non-significant for cancers (IRRs: 1.2–1.4). In general, associations are stronger for the placed group than for those investigated. Though IRRs for investigated women had similar directionality as for the placed women, this group was small with larger CIs that often include one (online supplemental table 4).

Table 3 shows the HRs of all-cause mortality after hospitalisation across ages 20–66. Experiences of ICWS were associated with increased risks of death after hospitalisation due to external causes (HRs: 1.7–2.6) and NCDs (HRs: 1.6–2.3), and, particularly, after hospitalisation due to CVDs (HRs: 1.8–2.5). Except for investigated women, ICWS was also associated with higher death hazards after hospitalisation due to mental and behavioural disorders (HRs: 1.5–2.0).

**Table 3:** The association between ICWS and all-cause mortality after hospitalisation, stratified by sex and cause group

	Men (n=7204)					Women (n=6930)				
	Observations/deaths	Investigated		Placed		Observations/deaths	Investigated		Placed	
		HR	95% CI	HR	95% CI		HR	95% CI	HR	95% CI
External causes	2459/533	**1.65**	1.34 to 2.02	**2.35**	1.87 to 2.96	1989/291	**2.21**	1.54 to 3.16	**2.55**	1.90 to 3.44
Self-harm	247/116	1.59	1.00 to 2.51	1.74	1.09 to 2.78	257/77	1.10	0.54 to 2.23	1.58	0.91 to 2.73
NCDs	5033/871	**1.69**	1.43 to 1.99	**2.33**	1.95 to 2.79	5064/567	**1.61**	1.21 to 2.15	**2.08**	1.66 to 2.62
Mental and behavioural disorders	1146/435	**1.48**	1.17 to 1.88	**1.56**	1.23 to 1.99	867/208	1.34	0.87 to 2.06	**2.00**	1.46 to 2.75
Anxiety	251/81	1.20	0.66 to 2.18	1.23	0.70 to 2.16	258/51	0.44	0.10 to 1.90	1.83	0.94 to 3.56
Depression	283/89	1.70	0.96 to 3.02	1.80	1.08 to 2.99	313/70	0.85	0.33 to 2.16	1.64	0.94 to 2.87
Substance use disorders	757/342	1.09	0.83 to 1.42	1.29	0.97 to 1.70	419/133	1.32	0.79 to 2.18	**2.03**	1.37 to 3.02
CVDs	2195/376	**1.75**	1.37 to 2.23	**2.49**	1.88 to 3.30	1530/225	**2.09**	1.39 to 3.14	**2.27**	1.59 to 3.25
Cancers	848/295	**1.56**	1.17 to 2.08	1.52	1.08 to 2.15	1094/309	1.38	0.90 to 2.11	1.47	1.01 to 2.13
Respiratory	816/198	1.22	0.86 to 1.73	**2.30**	1.58 to 3.35	814/151	1.13	0.61 to 2.11	1.63	1.09 to 2.43
Diabetes	381/100	1.40	0.86 to 2.29	**3.00**	1.74 to 5.17	243/57	1.97	0.86 to 4.52	1.65	0.80 to 3.38

Results from Cox proportional hazards models.

Reference group is ‘Without ICWS’. (HR=1.00). The models are centred around the first admission date and uses a calendar time scale. All models are adjusted for born out of wedlock, mother’s age at birth, occupational status of the father at birth, and family education at age 10. Bold numbers signify a p value ≤0.004.

CI, CI interval; CVDs, Cardiovascular diseases; HR, HR ratio; ICWS, involvement with child welfare services; NCDs, Non-communicable diseases.

## Discussion

Based on a 1953 birth cohort from Stockholm, followed over almost seven decades, our findings could largely confirm the initial hypotheses. Individuals with experiences of childhood adversity, by proxy of ICWS, had higher risks of being hospitalised in adulthood due to most of the major causes included here (differential risk of hospitalisation). Overall, they were also more likely to die after being hospitalised (differential survival). The results generally followed a gradient reflecting the severity of ICWS, which suggests that the results are driven by the underlying exposure to adversity rather than solely being due to placement in OHC itself. Moreover, there were no clear differences between men and women.

Extending the follow-up to mid-adulthood, our study corroborates findings from earlier studies showing that individuals with experiences of OHC have increased risks for hospitalisation due to external causes and mental and behavioural disorders—especially substance use disorders and self-harm.^14 16^ We further add evidence for elevated hospitalisation risks due to NCDs, and the subcategories of CVDs, respiratory diseases and diabetes. This is remarkable as such diseases typically have a later onset, and selective mortality among those with ICWS would be expected to bias these estimates towards the null. The investigation into subcategories also shows that the conditions most strongly related to health risk behaviours are particularly increased among those with experiences of OHC placement. This potentially indicates that such behaviours are perhaps used as strategy to cope with the increased burden of mental health problems in those with experiences of ICWS.

The findings also highlight that cancers are an exception, potentially because we could not differentiate the types of cancers with stronger behavioural risk factors. Our results are nonetheless in line with two of the most comprehensive earlier prospective inquiries into hospitalisation risks, which found a higher incidence across many conditions, but not neoplasms among individuals with a history of foster care.^28 30^ This is in contrast to other retrospective studies on cumulative adverse childhood experiences,^2^ and to two prospective studies finding evidence for increased cancer risks among women and lung cancer risks among those with childhood adversity.^34 35^

This study uniquely investigates the association between childhood adversity and survival prospects following hospitalisation. Individuals with experiences of ICWS generally had higher mortality risks and died sooner after hospitalisation due to external causes or NCDs than individuals without such experiences. These results were consistent in the subcategory of CVDs but not in the other subcategories. The worse survival prospects after a CVD-related hospitalisation could mean that individuals with experiences of ICWS are more vulnerable to the adverse consequences of such diseases, perhaps due to comorbidities, worse mental health or other coinciding risk factors for worse prognoses. Alternatively, it could reflect differences in disease severity when they present to the hospital, or differences in quality, access or response to treatment. These worse survival prospects might point to unmet care needs among individuals with experiences of childhood adversity; something which has been shown in OHC-experienced populations already during their childhoods.^36^

This study has several strengths. Most importantly, the population-based birth cohort data that could be followed up to age 66. Based on national registers, there was minimal attrition and selection. Some limitations should, however, be considered. We cannot exclude the possibility of measurement error. Our indicators of ICWS were based on child welfare records from the Social Registers kept locally by municipalities in Stockholm; not all cohort members lived in Stockholm before age 10 and we might miss some cases that were solely recorded outside the metropolitan area.^37^ In terms of hospitalisations, the Swedish National Patient Register did not have full national coverage between 1973 and 1987.^38^ However, the coverage in Stockholm was more or less complete (for a more extensive discussion, see Ludvigsson *et al*).

There are possible unmeasured confounders, such as baseline health, and other factors related to genetic predisposition to disease or risk-taking behaviours. There are also a number of potential mediators that have been not included in this study, for instance, life style behaviours, mental health, cognitive ability and educational attainment. Our results, nonetheless, lend some support to the hypothesis that mental and behavioural problems might be important explanatory factors. How much, for example, of the worse survival prospects, might be explained by mental health warrants further investigation: in patients with several comorbidities who experienced ICWS, mental health problems are very common (online supplemental table 5). Nevertheless, the cause groups used here were too broad and numerous to consider disease aetiology.

It remains a question whether our findings may explain previously reported inequalities in premature all-cause mortality between those with and without experiences of ICWS.^7^ Differential risk of hospitalisation and differential survival after hospitalisation have been considered important for producing socioeconomic inequalities in mortality more generally.^39^ Further investigating the mechanisms driving the inequalities in mortality following childhood adversity is a task for future research to consider.^40^

As this study only reports associations for one cohort born in one city, future research would need to verify the results in other contexts, and use designs and methods better equipped for making causal inferences. Regardless, the impact of adversity seems to persist across the life course, making it a relevant issue for public health and the inquiry into health inequalities. Our results further have implications for healthcare policy, as they strongly suggest that populations with experiences of childhood adversity might not be optimally served by the healthcare system, whether due to intrinsic susceptibility, help-seeking behaviours, comorbidity or other barriers. With their higher risks of disease and death following diseases, much could be gained from improving access to and quality of care in this vulnerable population.

## Data Availability

Under Swedish law and ethical approval, patient level data cannot be made publicly available. The statistical code is available from the corresponding author. Data access can be granted upon request to YBA and conditional on approval of the review board.

## References

[1] Hughes K , Ford K , Bellis MA , et al . Health and financial costs of adverse childhood experiences in 28 european countries: a systematic review and meta-analysis. Lancet Public Health 2021;6:e848–57. 10.1016/S2468-2667(21)00232-2 34756168 PMC8573710

[2] Hughes K , Bellis MA , Hardcastle KA , et al . The effect of multiple adverse childhood experiences on health: a systematic review and meta-analysis. Lancet Public Health 2017;2:e356–66. 10.1016/S2468-2667(17)30118-4 29253477

[3] World Health Organization Regional Office for Europe . Investing in children: the european child maltreatment prevention action plan 2015–2020. Copenhagen: WHO Regional Office for Europe, 2015.

[4] Bruskas D , Tessin DH . Adverse childhood experiences and psychosocial well-being of women who were in foster care as children. Perm J 2013;17:e131–41. 10.7812/TPP/12-121 24355905 PMC3783064

[5] Bonander C , Jernbro C . Does gender moderate the association between intellectual ability and accidental injuries? evidence from the 1953 stockholm birth cohort study. Accid Anal Prev 2017;106:109–14. 10.1016/j.aap.2017.06.001 28600987

[6] Murray ET , Lacey R , Maughan B , et al . Association of childhood out-of-home care status with all-cause mortality up to 42-years later: office of national statistics longitudinal study. BMC Public Health 2020;20:735. 10.1186/s12889-020-08867-3 32434479 PMC7238620

[7] Jackisch J , Brännström L , Almquist YB . Troubled childhoods cast long shadows: childhood adversity and premature all-cause mortality in a swedish cohort. SSM Popul Health 2019;9:100506. 10.1016/j.ssmph.2019.100506 31720363 PMC6838963

[8] Batty GD , Kivimäki M , Frank P . State care in childhood and adult mortality: a systematic review and meta-analysis of prospective cohort studies. Lancet Public Health 2022;7:e504–14. 10.1016/S2468-2667(22)00081-0 35660212 PMC9595443

[9] Soares S , Rocha V , Kelly-Irving M , et al . Adverse childhood events and health biomarkers: a systematic review. Front Public Health 2021;9:649825. 10.3389/fpubh.2021.649825 34490175 PMC8417002

[10] Barboza Solís C , Kelly-Irving M , Fantin R , et al . Adverse childhood experiences and physiological wear-and-tear in midlife: findings from the 1958 British birth cohort. Proc Natl Acad Sci U S A 2015;112:E738–46. 10.1073/pnas.1417325112 25646470 PMC4343178

[11] Krieger N . A glossary for social epidemiology. J Epidemiol Community Health 2001;55:693–700. 10.1136/jech.55.10.693 11553651 PMC1731785

[12] Gypen L , Vanderfaeillie J , De Maeyer S , et al . Outcomes of children who grew up in foster care: systematic-review. Children and Youth Services Review 2017;76:74–83. 10.1016/j.childyouth.2017.02.035

[13] McKenna S , Donnelly M , Onyeka IN , et al . Experience of child welfare services and long-term adult mental health outcomes: a scoping review. Soc Psychiatry Psychiatr Epidemiol 2021;56:1115–45. 10.1007/s00127-021-02069-x 33779782 PMC8225538

[14] Kääriälä A , Hiilamo H . Children in out-of-home care as young adults: a systematic review of outcomes in the Nordic countries. Children and Youth Services Review 2017;79:107–14. 10.1016/j.childyouth.2017.05.030

[15] Vinnerljung B , Hjern A . Consumption of psychotropic drugs among adults who were in societal care during their childhood-A Swedish national cohort study. Nord J Psychiatry 2014;68:611–9. 10.3109/08039488.2014.902501 24754468

[16] Vinnerljung B , Hjern A , Lindblad F . Suicide attempts and severe psychiatric morbidity among former child welfare clients -- a national cohort study. J Child Psychol Psychiatry 2006;47:723–33. 10.1111/j.1469-7610.2005.01530.x 16790007

[17] Almquist YB , Rojas Y , Vinnerljung B , et al . Association of child placement in out-of-home care with trajectories of hospitalization because of suicide attempts from early to late adulthood. JAMA Netw Open 2020;3:e206639. 10.1001/jamanetworkopen.2020.6639 32484554 PMC7267851

[18] Vinnerljung B , Sallnäs M . Into adulthood: a follow-up study of 718 young people who were placed in out-of-home care during their teens. Child Fam Soc Work 2008;13:144–55. 10.1111/j.1365-2206.2007.00527.x

[19] Sariaslan A , Kääriälä A , Pitkänen J , et al . Long-term health and social outcomes in children and adolescents placed in out-of-home care. JAMA Pediatr 2022;176:e214324. 10.1001/jamapediatrics.2021.4324 34694331 PMC8546624

[20] World Health Organization . The european health report 2021. taking stock of the health-related sustainable development goals in the COVID-19 era with a focus on leaving no one behind. Copenhagen: World Health Organization, 2022.

[21] Holman DM , Ports KA , Buchanan ND , et al . The association between adverse childhood experiences and risk of cancer in adulthood: a systematic review of the literature. Pediatrics 2016;138(Suppl 1):S81–91. 10.1542/peds.2015-4268L 27940981 PMC5892430

[22] Appleton AA , Holdsworth E , Ryan M , et al . Measuring childhood adversity in life course cardiovascular research: a systematic review. Psychosom Med 2017;79:434–40. 10.1097/PSY.0000000000000430 27893587

[23] Jakubowski KP , Cundiff JM , Matthews KA . Cumulative childhood adversity and adult cardiometabolic disease: a meta-analysis. Health Psychol 2018;37:701–15. 10.1037/hea0000637 30024227 PMC6109976

[24] Godoy LC , Frankfurter C , Cooper M , et al . Association of adverse childhood experiences with cardiovascular disease later in life: a review. JAMA Cardiol 2021;6:228–35. 10.1001/jamacardio.2020.6050 33263716

[25] Huang H , Yan P , Shan Z , et al . Adverse childhood experiences and risk of type 2 diabetes: a systematic review and meta-analysis. Metabolism 2015;64:1408–18. 10.1016/j.metabol.2015.08.019 26404480

[26] Petruccelli K , Davis J , Berman T . Adverse childhood experiences and associated health outcomes: a systematic review and meta-analysis. Child Abuse Negl 2019;97:104127. 10.1016/j.chiabu.2019.104127 31454589

[27] Grummitt LR , Kreski NT , Kim SG , et al . Association of childhood adversity with morbidity and mortality in US adults: a systematic review. JAMA Pediatr 2021;175:1269–78. 10.1001/jamapediatrics.2021.2320 34605870 PMC9059254

[28] Zlotnick C , Tam TW , Soman LA . Life course outcomes on mental and physical health: the impact of foster care on adulthood. Am J Public Health 2012;102:534–40. 10.2105/AJPH.2011.300285 22390519 PMC3487656

[29] Kelly-Irving M , Lepage B , Dedieu D , et al . Childhood adversity as a risk for cancer: findings from the 1958 British birth cohort study. BMC Public Health 2013;13:767. 10.1186/1471-2458-13-767 23957659 PMC3765119

[30] Rod NH , Bengtsson J , Elsenburg LK , et al . Hospitalisation patterns among children exposed to childhood adversity: a population-based cohort study of half a million children. Lancet Public Health 2021;6:e826–35. 10.1016/S2468-2667(21)00158-4 34599895

[31] VanderWeele TJ . Outcome-wide epidemiology. Epidemiology 2017;28:399–402. 10.1097/EDE.0000000000000641 28166102 PMC5378648

[32] Almquist YB , Grotta A , Vågerö D , et al . Cohort profile update: the stockholm birth cohort study. Int J Epidemiol 2019;63:1273–4. 10.1093/ije/dyz185 PMC7266536 31539027

[33] Forsman H , Jackisch J . Cumulative childhood adversity and long-term educational outcomes in individuals with out-of-home care experience: do multiples matter for a population defined by adversity? Br J Soc Work 2022;52:2495–514. 10.1093/bjsw/bcab194

[34] Berger E , Castagné R , Chadeau-Hyam M , et al . Multi-Cohort study identifies social determinants of systemic inflammation over the life course. Nat Commun 2019;10:773.:773. 10.1038/s41467-019-08732-x 30770820 PMC6377676

[35] Brown DW , Anda RF , Felitti VJ , et al . Adverse childhood experiences are associated with the risk of lung cancer: a prospective cohort study. BMC Public Health 2010;10:20. 10.1186/1471-2458-10-20 20085623 PMC2826284

[36] Vinnerljung B , Hjern A . Health and health care for children in out‐of‐home care. Int J Soc Welfare 2018;27:321–4. 10.1111/ijsw.12352 Available: https://onlinelibrary.wiley.com/toc/14682397/27/4

[37] Jackisch J . *Troubled childhoods cast long shadows: studies of childhood adversity and premature mortality in a swedish post-war birth cohort (dissertation)* . Stockholm, Stockholm University, 2022

[38] Ludvigsson JF , Andersson E , Ekbom A , et al . External review and validation of the Swedish national inpatient register. BMC Public Health 2011;11:450. 10.1186/1471-2458-11-450 21658213 PMC3142234

[39] Diderichsen F , Evans T , Whitehead M . The social basis of disparities in health. In: Challenging Inequities in Health: From Ethics to Action. 2001: 12–23.

[40] Diderichsen F , Hallqvist J , Whitehead M . Differential vulnerability and susceptibility: how to make use of recent development in our understanding of mediation and interaction to tackle health inequalities. Int J Epidemiol 2019;48:268–74. 10.1093/ije/dyy167 30085114

