# Supplementary material for: Childhood adversity is associated with hospitalisations and survival following external causes and non-communicable diseases: a 46-year follow-up of a Stockholm birth cohort
Source: J Epidemiol Community Health. 2023 Feb 3;77(4):209–15. doi: 10.1136/jech-2022-219851 (PMC10086507; doi:10.1136/jech-2022-219851)
Supplement: Supplementary data [file jech-2022-219851supp001.pdf]

Supplementary Material

Supplementary Table 1. ICD (10, 9, 8) codes included in each disease group.

Disease group	ICD 10 (1997-2020)	ICD 9 (1987-1996)	ICD 8 (1973-1986)
External causes (excl. alcohol related)	E000-E999, V01-Y89 (excl. X41-X42, X44, X45)	E000-E999	E000-E999
Self-harm[1,2,3,4]	X60-X84, Y870, Y10-Y34	E950-E959, E980-E989	E950-E959, E980-E989
NCDs (non-communicable diseases)	C00-C97, D00-D48, D55-D64 (excl. D 64.9), D65-D89, E03-E34, E65-E88, F01-F99, G06-G98 (excl. G14), H00-H61, H68-H93, I00-I99, J30-J98, K00-K92, L00-L98, M00-M99, N00-N64, N75-N98, Q00-Q99, R95	140-242, 244-259, 270-279 (excl. 279.5), 282-285 (excl. 285.9), 290-319, 324-380, 383-459, 470-478, 490-611, 617-629, 680-759	140-209, 230-239, 244-258, 270-279, 282-285, 290-315, 321, 322, 324, 330-380, 383-459, 460-519, 520-611, 623-629, 680-759
Mental and behavioural disorders	F04-F99, G72.1, Q86.0, X41-X42, X44, X45	2900-319, 324-359, 7598, 7800, E851-E852, E860	290-315
Anxiety[5]	F40-F44	300.A, 300.B, 300.C, 300.D, 308.A, 308.B, 308.D, 308.E, 308.X, 309.W	299, 300.0, 300.1, 300.2, 300.3, 306.9, 301.8, 307, 308, 781.5
Depression[5]	F32-F33, F34.1	296.B, 296.G, 296.X, 298.A, 304.E, 311	296.0, 296.1, 296.2, 296.8, 296.9, 298.0, 300.0, 300.4, 304.4, 790.2
Substance use disorders[6]	E24.4, F10-F16, F18-F19, I42.6, G31.2, G72.1, Q86.0, R78.0, T40, T42.3-T42.6, T43.6, X41-X42, X44d, X45, Y15, Z50.2, Z50.3 Z71.4, Z71.5 Z72.1, Z72.2	255, 291,292, 303, 304, 305, 357.F, 425.F, 571, 790.3, 965.A, 968.E, 969G-H, 975.C, 980, V578, V654, V658, Y39, Y79, E860	2580, 2589, 291, 2943, 303, 304 57100, 962.0, 966.x, 967.0, 967.9, 969, 976.9, 970, 971, 980, E860
CVDs (cardiovascular diseases)	I00-I99	390-459	390-459
Cancers (malignant neoplasms)	C00-C97	140-208, 230-239	140-209, 230-239
Respiratory diseases (chronic respiratory diseases)	J30-J98	470-4789, 490-496, 500-509, 5110-5198, 6800-6820, 7309-7339	460-519
Diabetes	E10-E14 (excl. E10.2, E11.2, E12.2, E13.2, E14.2)	2500-2510 (excl. 2503)	249-250

The categorisation above mostly follows the general categorisation used in the Global Burden of Disease studies: http://www.healthdata.org/sites/default/files/files/data_for_download/2012/IHME_GBD2010_CauseListandICD.pdf

References

1. Vinnerljung B, Hjern A, Lindblad F. Suicide attempts and severe psychiatric morbidity among former child welfare clients–a national cohort study. Journal of child psychology and psychiatry. 2006;47(7):723-733.
2. Björkenstam C, Björkenstam E, Ljung R, Vinnerljung B, Tuvblad C. Suicidal behavior among delinquent former child welfare clients. European child & adolescent psychiatry. 2013;22(6):349-355.
3. Björkenstam C, Kosidou K, Björkenstam E. Childhood adversity and risk of suicide: cohort study of 548 721 adolescents and young adults in Sweden. BMJ. 2017;357:j1334.
4. Björkenstam C. Epidemiological studies of suicide: classification bias, drug use, and social circumstances. Inst för folkhälsovetenskap/Dept of Public Health Sciences; 2013.
5. Modin B, Östberg V, Almquist Y. Childhood peer status and adult susceptibility to anxiety and depression. A 30-year follow-up of Stockholm girls. Journal of Abnormal Child Psychology. 2011;39(2):187-99.
6. Following the outcome operationalisation used in the same sample by Bishop & Almquist, 2021; Friends' childhood adversity and long-term implications for substance misuse: a prospective Swedish cohort study. Addiction;116: 632-640.

Supplementary Table 2. Model fit statistics for the count variable of hospitalisations due to major cause groups, covariate-adjusted poisson, zero-inflated poisson, and negative binomial models

	BIC			AIC		
	Poisson	Zero-inflated poisson	Negative binomial	Poisson	Zero-inflated poisson	Negative binomial
External	36929.527	32441.323	29834.324	36853.964	32290.197	29751.205
Self-harm	8210.633	5987.518	5697.830	8135.070	5836.391	5614.710
NCDs	140225.876	120826.591	67992.480	140203.207	120781.253	67962.255
Mental and behavioural disorders	86777.671	43985.199	22243.975	86702.107	43834.072	22160.856
Anxiety	12217.546	7399.523	6117.783	12141.982	7248.396	6034.663
Depression	12454.032	7732.637	6956.307	12378.469	7581.510	6873.188
Substance misuse	57373.546	27069.904	14173.146	57297.983	14090.026	26918.778
CVDs	43523.547	33640.796	28874.652	43447.984	33489.669	28791.533
Cancer	36145.042	22295.978	18926.588	36069.478	22144.851	18843.468
Respiratory	19894.914	15988.411	14231.532	19819.351	15837.284	14148.412
Diabetes	18113.459	9895.384	7543.627	18037.895	9744.258	7460.507

Note: All models are adjusted for born out of wedlock, mother's age at birth, occupational status of the father at birth, and family education at age 10.

BMJ Publishing Group Limited (BMJ) disclaims all liability and responsibility arising from any reliance placed on this supplemental material which has been supplied by the author(s)

Additional background on hospitalisations

Of the over 78,000 hospitalisations, 75% were due to NCDs, and 12% were due to external causes. The most prevalent primary and secondary causes of hospitalisations (except pregnancy) were external causes, diseases of the digestive system, diseases of the genitourinary system, cancers, musculoskeletal diseases, CVDs, respiratory diseases, and mental and behavioural disorders. The most important causes of deaths between ages 20-66 were cancers (35%), CVDs (19%), external causes (18%), diseases of the digestive system (6%), mental and behavioural disorders (4%), and disease of the respiratory system (4%).

Supplementary Table 3. Hospitalisations in ages 20-66: Median number of hospitalisations (frequency) and the median age at first hospitalisation (onset), among those with at least one hospitalisation within each disease group, stratified by sex and ICWS group.

	**Hospitalisations among males in ages 20-66 (n=7,204)**						**Hospitalisations among females in ages 20-66 (n=6,930)**					
	**Without ICWS**		**Investigated**		**Placed**		**Without ICWS**		**Investigated**		**Placed**	
	Frequency	Onset	Frequency	Onset	Frequency	Onset	Frequency	Onset	Frequency	Onset	Frequency	Onset
External causes (n=2,472)	1	41.5	2	36.7	2	33.0	1	48.2	1	45.4	2	42.8
Self-harm (n=250)	1	35.9	1	35.3	1	30.2	1	37.1	1	35.0	1	38.4
NCDs (n=5,042)	3	41.6	3	40.1	5	32.3	3	36.6	3	30.7	4	30.6
Mental and behavioural disorders (n=1,146)	2	41.2	3	40.1	5	32.0	2	40.0	3	31.0	4	32.3
Anxiety (n=251)	1	45.4	1	45.8	2	30.1	1	46.7	2	48.3	1	29.3
Depression (n=283)	1	42.7	1	44.3	2	35.5	1	47.1	1	33.4	2	43.7
Substance use disorders (n=757)	2	46.6	3	39.2	5	31.6	2	47.0	2	46.5	4	45.5
CVDs (n=2,207)	2	57.6	2	56.8	2	56.9	1	56.9	1	56.8	1	56.1
Cancers (n=850)	2	60.2	2	59.8	2	61.2	2	54.2	2	56.7	3	52.0
Respiratory diseases (n=821)	1	33.4	1	41.0	1	34.9	1	34.2	1	33.7	1	39.6
Diabetes (n=381)	2	58.0	2	57.0	2	55.3	2	55.1	2	57.2	3	55.4

Supplementary Table 4. The association between ICWS and disease-group-specific hospitalisations, stratified by sex. Results from negative binomial regression.

	Males (N=7204)				Female (N=6930)			
	**Investigated**		**Placed**		**Investigated**		**Placed**	
	**IRR**	**95% CI**	**IRR**	**95% CI**	**IRR**	**95% CI**	**IRR**	**95% CI**
External	**1.69**	1.51,1.90	**2.21**	1.91,2.55	**1.40**	1.14,1.73	**1.94**	1.62,2.32
Self-harm	**2.62**	1.77,3.86	**4.73**	2.95,7.57	**2.06**	1.12,3.78	**4.11**	2.45,6.89
NCDs	**1.66**	1.51,1.82	**2.69**	2.40,3.03	**1.43**	1.25,1.64	**2.20**	1.95,2.47
Mental and behavioural disorders	**2.71**	2.07,3.54	**6.73**	4.83,9.38	**2.38**	1.50,3.78	**5.12**	3.41,7.70
Anxiety	**2.42**	1.54,3.82	**5.18**	3.00,8.95	1.51	0.74,3.08	**3.77**	2.05,6.93
Depression	1.79	1.15,2.81	**5.16**	3.09,8.62	1.04	0.55,1.98	**4.16**	2.45,7.07
Substance misuse	**3.96**	2.86,5.48	**9.94**	6.66,14.82	**5.44**	2.92,10.14	**10.72**	6.18,18.61
CVDs	**1.49**	1.29,1.72	1.30	1.08,1.56	**1.56**	1.21,2.02	1.35	1.08,1.70
Cancer	1.16	0.72,1.52	1.35	0.96,1.89	1.23	0.86,1.77	1.36	0.99,1.87
Respiratory	**1.46**	1.17,1.84	**2.34**	1.77,3.09	1.25	0.88,1.77	**2.31**	1.73,3.09
Diabetes	**2.00**	1.37,2.93	1.63	0.99,2.66	1.96	0.84,4.55	**3.04**	1.46,6.33

Notes: NCDs=Non-communicable diseases; CVDs=Cardiovascular diseases; IRR=Incidence rate ratio; CI=Confidence interval. Reference group is 'Without ICWS'. (IRR=1.00). All models are adjusted for born out of wedlock, mother's age at birth, occupational status of the father at birth, and family education at age 10. Bold numbers signify a p-value <=0.004.

Supplementary Table 5. Prevalence of comorbidity between mental and behavioural disorders, and a count of cardiovascular diseases, cancers, respiratory diseases, diabetes, and external causes (n=14,134).

	Among those hospitalised due to mental and behavioural disorders								
	Without ICWS			Investigated			Placed		
Count of cardiovascular diseases, cancers, respiratory diseases, diabetes, and external causes	n	Column %	Prevalence of mental and behavioural disorder in this group (%)	n	Column %	Prevalence of mental and behavioural disorder in this group (%)	n	Column %	Prevalence of mental and behavioural disorder in this group (%)
0	289	24.1	5.5	52	13.6	9.3	74	17.0	17.5
1	423	35.3	11.8	138	36.1	24.0	157	36.1	35.1
2	310	25.9	18.0	119	31.2	33.2	125	28.7	46.6
3 or more	175	14.6	26.4	73	19.1	46.5	79	18.2	53.4
